# Lattice strain across Na–K interdiffusion fronts in alkali feldspar: an electron back-scatter diffraction study

**DOI:** 10.1007/s00269-014-0692-y

**Published:** 2014-06-27

**Authors:** Anne-Kathrin Schäffer, Tom Jäpel, Stefan Zaefferer, Rainer Abart, Dieter Rhede

**Affiliations:** 1 Department of Lithospheric Research, University of Vienna, Althanstrasse 14, 1090 Vienna, Austria; 2 Max-Planck Institute for Iron Research, Max-Planck-Strasse 1, 40237 Düsseldorf, Germany; 3 Helmholtzzentrum Potsdam, Deutsches GeoForschungsZentrum, Telegrafenberg, 14473 Potsdam, Germany

**Keywords:** Alkali feldspar, Interdiffusion, Coherency strain, EBSD

## Abstract

Cation exchange experiments between gem quality sanidine $(X_\mathrm{Or} = 0.85)$ and KCl melt produced chemical alteration of alkali feldspar starting at the grain surface and propagating inwards by highly anisotropic Na–K interdiffusion on the alkali sublattice. Diffusion fronts developing in *b*-direction are very sharp, while diffusion fronts within the *a*–*c*-plane are comparatively broad. Due to the composition dependence of the lattice parameters of alkali feldspar, the diffusion induced compositional heterogeneity induces coherency stress and elastic strain. Electron back-scatter diffraction combined with the cross-correlation technique was employed to determine the lattice strain distribution across the Na–K interdiffusion fronts in partially exchanged single crystals of alkali feldspar. The strain changes gradually across the broad fronts within the *a*–*c*-plane, with a successive extension primarily in *a*-direction conferring to the composition strain in unstressed alkali feldspar. In contrast, lattice strain characterised by pronounced extension in *b*-direction is localised at the sharp diffusion fronts parallel to *b*, followed by a slight expansion in *a*-direction in the orthoclase-rich rim. This strain pattern does not confer with the composition induced lattice strain in a stress-free alkali feldspar. It may rather be explained by the mechanical coupling of the exchanged surface layer and the mechanically strong substratum. The lattice distortion localised at the sharp diffusion front may have an influence on the diffusion process and appears to produce a self-sharpening feedback, leading to a local reduction of component mobilities.

## Introduction

Alkali feldspars are among the most abundant rock-forming minerals in the Earth’s crust. They pertain to the binary solid-solution series comprising the end-member components orthoclase $(\hbox {KAlSi}_3{\mathrm{O}}_8)$ and albite $(\hbox {NaAlSi}_3{\mathrm{O}}_8)$. Alkali feldspars occur in magmatic, metamorphic and sedimentary environments. Mineral parageneses involving alkali feldspar, and in particular the chemical compositions and zoning patterns of alkali feldspar, may bear important petrogenetic information (Spear 1993). In this context, the robustness of chemical compositions during prolonged thermal annealing or metamorphic overprint is of crucial interest.

Chemical alteration of alkali feldspar in the solid state requires the interdiffusion of $\hbox {Na}^+$and $\hbox {K}^+$ and potentially substituting minor components on the respective sublattice. Na–K interdiffusion is relatively rapid (Cherniak 2010), and Na–K cation exchange reactions involving alkali feldspar, such as the two feldspar thermometer (Benisek et al. 2004), and the compositions of the albite- and orthoclase-rich phases in a perthite (Yund 1984; Abart et al. 2009; Petrishcheva and Abart 2012) are prone to re-equilibration during slow cooling. Quantification of these potential effects requires knowledge of the Na–K interdiffusion coefficient in alkali feldspar. Among others, this motivated cation exchange experiments (Petrović 1972; Neusser et al. 2012; Petrishcheva et al. submitted; Schäffer et al. submitted) and diffusion couple experiments (Christoffersen et al. 1983), which delivered composition-distance data from which the interdiffusion coefficient can be extracted. Complications may arise during interdiffusion experiments due to the composition dependence of the lattice parameters of alkali feldspar.

The crystal structure of alkali feldspar is comprised of an aluminosilicate framework where corner-sharing $\hbox {AlO}_4$- and $\hbox {SiO}_4$-tetrahedra are linked in a three-dimensional network forming crankshaft-like chains parallel to the *a*-axis. Cavities in the tetrahedral framework are occupied by the $\hbox {Na}^+$ and $\hbox {K}^+$ cations with minor substitution by $\hbox {Rb}^+,\, \hbox {Ca}^{2+}$, and $\hbox {Ba}^{2+}$ (Ribbe 1983). Incorporation of differently sized cations is mainly accommodated by the stretch of the crankshaft-like chains parallel to the *a*-axis (Petrović 1972; Angel et al. 2012). This leads to a pronounced anisotropy of the composition dependence of the lattice parameters which is about five times higher in the *a*-direction than in the *b*- and *c*-directions (Kroll et al. 1986; Angel et al. 2012). Thus, any compositional heterogeneity in alkali feldspar, such as a chemically altered surface layer produced by cation exchange, causes coherency stress. The lattice contraction associated with a composition shift towards more sodium-rich composition may produce tensile (mode I) cracks (Petrović 1972; Neusser et al. 2012; Scheidl et al. 2013).

In contrast, if alkali feldspar of intermediate composition is shifted towards more potassium-rich compositions, this typically produces a potassium-rich surface layer, which is separated from the internal portions of the feldspar grain by a more or less sharp composition front, which propagates into the feldspar with time. If the composition shift across this front exceeds about 15 mole %, this may produce cracks, which tend to follow the exchange fronts. The fronts are exceptionally sharp in *b*-direction and broader in directions lying within the *a*–*c* plane. Petrishcheva et al. (submitted) and Schäffer et al. (submitted) produced surface layers with $X_\mathrm{Or} = 1.00$ on sanidine with an original composition of $X_\mathrm{Or} = 0.85$ by cation exchange with a KCl salt melt at 850 °C and ~1 bar. The composition fronts in *b*-direction are less than about 2 μm wide. In a stress-free alkali feldspar, a composition shift from $X_\mathrm{Or} = 0.85$ to $X_\mathrm{Or}$ = 1.00 corresponds to a change in lattice parameters of about 1 % in the *a*-direction and 0.2 % in the *b*- and *c*-directions. If the lattice is coherent across the composition front, this induces substantial stress and elastic strain. If the coherency stress exceeds a critical level, this may induce fracturing. The formation of cracks along the composition fronts in alkali feldspar that was treated in our experiments provides evidence for such a mechanism. In this communication, we focus on situations where fracturing did not occur. In such cases, the coherency strain is accommodated by elastic deformation. We quantify the coherency strain across composition fronts by direct measurement. To this end, we employ electron back-scatter diffraction (EBSD) measurements combined with the cross-correlation method to obtain the lattice strain along profiles taken across such composition fronts. The full strain tensor is obtained, and the systematics of the lattice strain is analysed in the light of the mechanical boundary conditions. Implications of a potential feedback between Na–K interdiffusion, diffusion induced coherency strain and associated distortion of the crystal lattice are discussed.

## Methods

### Cation exchange experiments

**Fig. 1:**
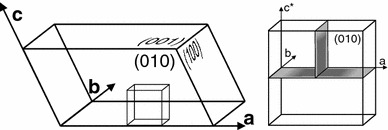
Sample geometry of the prepared plates; *left* orientation of the plate within the unit cell of feldspar, *right* after the experiment the plate was cut in two mutually perpendicular directions (*grey planes*) to allow documentation of interdiffusion fronts in three different crystallographic directions, i.e. *a*, *b* as well as c*, which is the direction perpendicular to the *a*–*b* plane and has an angle of about 26° to the *c*-direction

Cation exchange experiments were done using alkali feldspar and NaCl–KCl salt mixtures. Gem quality sanidine from Volkesfeld (Eifel) with an initial composition of $\hbox {X}_{\mathrm{Or}} = 0.85$ and minor Ba contents of up to 1 wt% BaO, (0.01 apfu) and Fe contents of up to 0.2 wt% FeO (0.01 apfu) was used as starting material. The sanidine is chemically homogeneous and devoid of cracks, twins, exsolutions, second phase precipitates or other structural or chemical heterogeneities (Riley and Bailey 2003; Parsons and Lee 2005; Weitz 1972; Neusser et al. 2012; Demtröder 2011). It has a monoclinic symmetry and crystallises in the space group C2/m. Aluminium and silicon on the tetrahedral site are highly disordered (*t*1 = 61) (Neusser et al. 2012). Plates of 3 × 3 mm size and 1 mm thickness were prepared. They were pre-oriented on the basis of their optical extinction under polarised light. Plates with polished (010) surfaces were produced (Fig. 1).

The crystallographically oriented plates were sealed in quartz glass tubes under vacuum together with a mixture of KCl and NaCl salts. The K over Na ratio of the salt mixture was chosen according to the experimentally determined Na–K partitioning between crystal and alkali chloride melt (Neusser et al. 2012) so that specific composition shifts were obtained. To ensure constant concentration boundary conditions during the exchange experiment, the amounts of salt and feldspar were chosen so that a 40:1 molar proportion of the alkali cations contained in the salt relative to those contained in the feldspar was obtained. The quartz glass tubes were then placed in a pre-heated muffle furnace and kept at constant temperatures ranging from 800 to 1,000 °C for 1 to 64 days. Temperatures were accurate within ±1 °C. At the end of the experiment, the melt was quenched within seconds by dropping the quartz glass tubes into cold water. The feldspar was retrieved by dissolving the salt with deionised water at room temperature. The crystals were subsequently cut in two mutually perpendicular directions normal to the polished surface (Fig. 1) to allow for the measurement of chemical profiles along three crystallographic directions. They were mounted in epoxy resin, first polished mechanically and then chemically using a colloidal silica suspension with a pH of 9.2–10.

### Mineral chemical analysis

Mineral chemical analysis was carried out at the Helmholtz Zentrum Potsdam, GFZ German Research Centre for Geosciences, using a JEOL Hyperprobe JXA-8500F with a thermal field emission cathode. The instrument was operated at an accelerating voltage of 8 kV and a beam current of 10 nA. For calibration, the beam was defocused to 10 μm, and for measurements the beam was fully focused. Peak measurements were set to 10 s and background measurements to 5 s. The relatively short counting time was necessary to minimise loss of alkali cations by evaporation during the measurement. The small excitation volume makes the measurements sensitive to imperfections and contamination of the sample surface and leads to some scattering in the data. Profiles were measured with a spot size of about 0.5 μm and a step size of 0.5 or 1 μm, depending on the length of the diffusion profile. The relative error is within 3.5–4 % for potassium and 7–8 % for sodium.

### Electron microscopy

Back-scattered electron (BSE) imaging was done using an FEI InspectS scanning electron microscope equipped with a tungsten filament. The instrument was operated at acceleration voltages of 10 and 15 kV and a beam current of 8 nA. The surface of the sample was coated with carbon using carbon evaporation of a double-thread of carbon fibre at a distance of about 8 cm from the sample under vacuum of 10^−5^ mbar during evaporation to establish electrical conductivity.

Electron back-scatter diffraction (EBSD) measurements were performed at the Max-Planck Institute for Iron Research in Düsseldorf using a Zeiss XB 1540 cross beam instrument with thermal field emission gun operated at 15 kV and about 5 nA beam current. Conductivity of the sample was established by a carbon sputter coating of 2–3 nm thickness applied with a Gatan PECS precision etching and coating instrument. The sample was mounted at 70° sample tilt, and the measurements were done at a working distance of 13 mm.

Profiles were measured parallel to the *b*- and c*-directions of the crystal; the sample was oriented so that the trace of the crystal surface was parallel to the tilt axis. To minimise beam damage on the beam sensitive feldspar, patterns were collected by scanning the beam over a rectangular area of 1 × 50 μm^2^ with its long axis parallel to the trace of the chemical gradient. This window was moved in 1 μm steps for profiles parallel to the *b*-direction and in 2 μm steps for profiles parallel to c*.

The OIM Data Collection software 5.3 was used for data acquisition. A TSL Hikari EBSD detector with a 640 × 640 pixel resolution was used for pattern recording. Patterns were recorded at 1 × 1 binning and subsequently corrected by subtracting the measured background, histogram normalisation, and applying a dynamic background subtraction. The background pattern was taken on a carbon-coated glass sample mounted on the same sample holder. For each pattern, the camera exposition time was 200 ms and 40 patterns were averaged, resulting in a total exposure time of about 8 seconds. For orientation determination, the patterns were binned to a pattern size of 240 pixels and a Hough transform with an angular resolution of 0.25 2*θ* step size was applied. The Hough transform was convoluted with a 13 × 13 pixel large convolution mask, and 20 peaks were identified with a minimum peak distance of 16 pixels. Only the inner part of the pattern was considered with the $\rho$ fraction of the Hough space being set to 84 %.

### EBSD cross-correlation technique

The acquired EBSD patterns (EBSP) were used to determine the full elastic strain tensor employing a pattern cross-correlation technique implemented in the commercial software CrossCourt 3 by BLG Productions. This technique is based on the measurement of pattern distortion by comparing the current pattern with a reference pattern obtained at a region which serves as a starting point for lattice strain determination. It is important that all acquisition parameters are kept constant during measurement of the reference and current pattern, and that the relative positions of the reference area and the measurement area are well known.

In the present case, the chemically altered rim is thin compared to the bulk of the sample. The internal region of the crystal is therefore mechanically much stronger than the thin chemically altered surface layer. As in the interior portions, the original composition is preserved and the crystal lattice can be assumed to be practically undeformed in the grain interior. Accordingly, the pattern obtained at the inner end of the profile in the chemically unaltered portion of the crystal was chosen as reference pattern, representing the original, unstrained lattice. Shifts of the EBSPs’ features in the regions of interest of each pattern obtained along the measured profile further outwards and across the sharp composition front were then compared to their equivalent in the reference pattern. Variations in these shifts across the pattern give insight into the nature of the strain within the diffracting volume (Wilkinson et al. 2009).

The analysis of pattern distortion was performed on the basis of 253 square regions of interest (ROI) each with 64 × 64 pixel size placed on the recorded EBSP in a regular grid within the circular area of the pattern with an overlap area of 7.5 px^2^ between neighbouring regions of interest. The sensitivity of the cross-correlation method strongly depends on pattern quality. For very good patterns, shifts as small as a few hundredths of pixels can be determined (Wilkinson and Britton 2012); such a high quality can unfortunately not be achieved for feldspars due to the necessity for a carbon coating of the surface, the low symmetry and density of the crystal as well as its low stability under the electron beam. Furthermore, as the patterns were taken by integration over an approximately 50 μm^2^ large area, they contain an inherent horizontal blurring of about 0.5 pixels (pixel size is 60 μm). Nevertheless, the pattern quality obtained in our measurements was sufficient to reach a sensitivity of about 0.5 pixels (compare Wilkinson et al. 2006). The strain sensitivity for high-resolution Kikuchi patterns is 1.3 × 10^−4^ (Wilkinson et al. 2006).

Fast Fourier filters were employed on the ROIs in order to reduce high-frequency intensity noise and low-frequency intensity background influences. With this the translational shifts in the tested pattern ROIs can be tracked more accurately by the cross correlation in the fourier domain (Wilkinson et al. 2009). The low-frequency cut-off was set to 2 px^−1^ with a cut-off width of 0, while the high-frequency cut-off was set to 15px^−1^, also with a cut-off width of 0.

## Results

### Chemical patterns from cation exchange

**Fig. 2:**
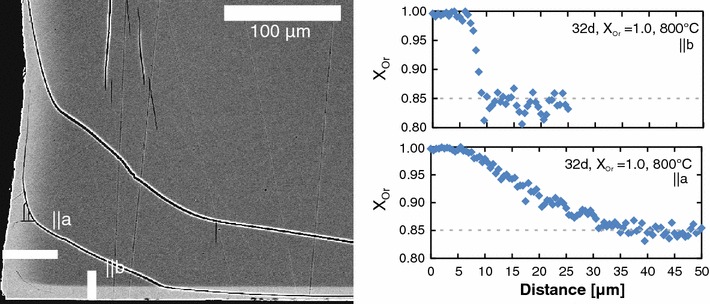
(001)-Section of a feldspar plate prepared with polished (010) surfaces and exchanged with pure KCl at 850 °C for 32 days; the BSE image shows bright areas along the grain surfaces which were in equilibrium with the melt, separated from darker areas of unexchanged feldspar in the core (*left*) by clearly anisotropic diffusion fronts; FEG-EMP measurements (*right*) along the profiles indicated in the BSE image show that even though the diffusion profiles vary in sharpness and width, they both exhibit the same shape, characterised by two plateaus and an inflection point of the diffusion front

Figure 2 shows a BSE image of alkali feldspar with an original $\hbox {X}_{\mathrm{Or}}$ of 0.85, which was reacted with pure KCl salt melt at 850 °C for 32 days. The bright areas along the grain surfaces represent the K-rich surface layer, which is separated from the dark grey internal regions of the grain by more or less sharp composition fronts. Cation exchange in the single crystal necessitates Na–K interdiffusion, and the composition fronts may be interpreted as diffusion fronts, which propagate into the crystal with time. The shape of the diffusion fronts, and in particular their sharpness, exhibits clear direction dependence. The widest diffusion fronts are observed in directions lying in the *a*–*c* plane, while the narrowest fronts develop in *b*-direction. The chemical profiles measured with a field emission gun-electron microprobe (FEG-EMP) across the diffusion fronts confirm this observation. It is important to note that the profiles exhibit two plateaus corresponding to the exchanged rim and the core in which the original composition is preserved, respectively, and the diffusion front between the two plateaus exhibits an inflection point (Fig. 2).

In experiments using salt melts with different compositions $(\hbox {X}_{\mathrm{KCl}}$ 0.6, 0.85 and 1.0), it was found that, apart from crystallographic direction, the sharpness of the diffusion front also depends on the extent of the chemical shift, i.e. the composition difference between the exchanged rim and unexchanged core. For a composition shift of only 5 mole-% to $X_\mathrm{Or} = 0.90$ the front is very indistinct and its geometry confers to what is expected for interdiffusion with constant concentration boundary conditions and constant interdiffusion coefficient. For a shift of 10 mole-% to a composition of $X_\mathrm{Or} = 0.95$ the plateaus begin to form and the front with the inflection point becomes apparent (Petrishcheva et al. submitted; Schäffer et al. submitted). Only if the composition is shifted to values of $X_\mathrm{Or}$ larger than 0.95, the described characteristic shape with its two sharply defined plateaus separated by a very narrow front develops.

**Fig. 3:**
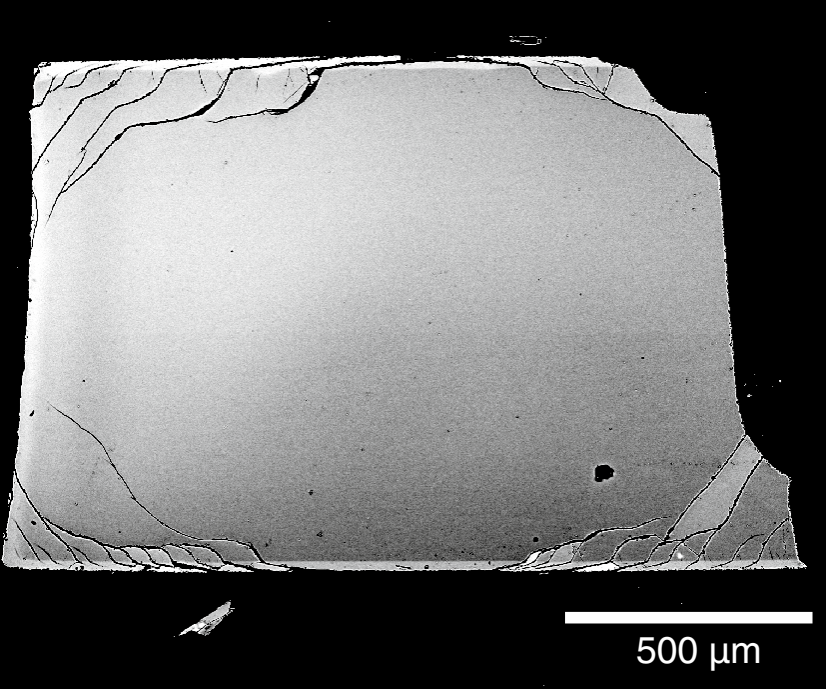
(001)-Section of a feldspar plate with polished (010) surfaces exchanged with pure KCl at 920 °C for 2 days; feldspars shifted to pure orthoclase composition often show complex crack patterns with cracks running roughly parallel to the interdiffusion fronts, caused by the anisotropy of the composition dependence of the lattice parameters

A composition shift from an initial $X_\mathrm{Or} = 0.85$ to pure orthoclase composition often leads to the formation of complex crack patterns (Fig. 3). Cracks only appear for shifts of more than about 12 mole-%. Run duration and temperature may also influence the crack pattern as they determine how far the diffusion fronts propagate into the crystal. The observed cracks run roughly parallel to the diffusion fronts. At the edges of the crystal plate, the cracks from two adjacent faces link up. Often the cracks form within yet unexchanged portions of the crystal slightly beneath the diffusion front. Most of the longer cracks run parallel to the *a*-axis of the feldspar, while they are usually much shorter parallel to *b*- and c*. This is related to the anisotropy of the composition dependence of the lattice parameters of feldspar, which is more pronounced parallel to the *a*-axis than in any other direction. A detailed mechanical analysis of the crack pattern is beyond the purpose of this communication. Nevertheless, the cracks may be seen as an indication for the build-up of coherency stress during cation exchange.

### Lattice strain across diffusion fronts

**Fig. 4:**
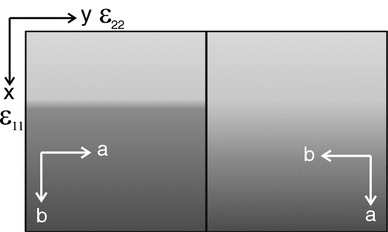
Relation between lattice direction and instrumental reference frame for samples with sharp diffusion fronts (*left*) and broad diffusion fonts (*right*)

In cases where the shifts towards more potassium-rich compositions do not induce fracturing, the lattice misfit across the diffusion fronts is accommodated by elastic strain. The associated lattice distortion was investigated by EBSD combined with the cross-correlation method. Lattice strain across wide and sharp diffusion fronts is treated separately. It must be noted that the samples were in different orientation with respect to the instrumental reference frame for the analysis of lattice strain across the sharp and broad fronts (Fig. 4). For the analysis of the sharp fronts, the longitudinal strain $\epsilon _{11}$ corresponds to the *b*-direction, and $\epsilon _{22}$ corresponds to the *a* direction. In contrast, for the analysis of broad fronts, the longitudinal strain $\epsilon _{11}$ corresponds to the *a*-direction, and $\epsilon _{22}$ corresponds to the *b*-direction.

**Fig. 5:**
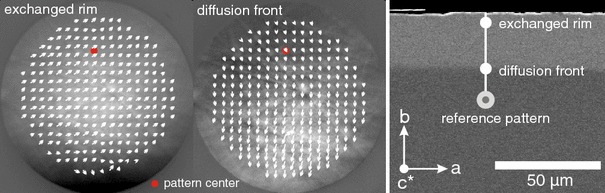
EBSP with shifts relative to the reference pattern indicated by the *white arrows* (*arrows* scaled to ten); in the exchanged rim, the shifts have a major component parallel to the *a*-direction of the crystal, while in the diffusion front itself, a predominant shift parallel to the *b*-axis is observed

The sample chosen for analysis of lattice strain across a sharp diffusion front was polished on the (010) surface and then exchanged with pure KCl melt for 4 days at 920 °C. A sharp diffusion front developed inwards from the polished (010) surface, i.e. in *b*-direction. EBSD patterns were collected along several profiles, which start in the unexchanged grain interior, run across the sharp diffusion front and end at the polished surface. The patterns in the exchanged rim and across the diffusion front are clearly distorted relative to the reference pattern which was taken at the inner end of the profile where the original composition of the feldspar is preserved. The shifts of the patterns recorded in the rim relative to the reference pattern have the largest component in the *a*-direction, indicating that the largest distortion of the crystal occurs in this direction. Localised at the diffusion front itself a major component parallel to the *b*-direction of the crystal is documented (Fig. 5).

**Fig. 6:**
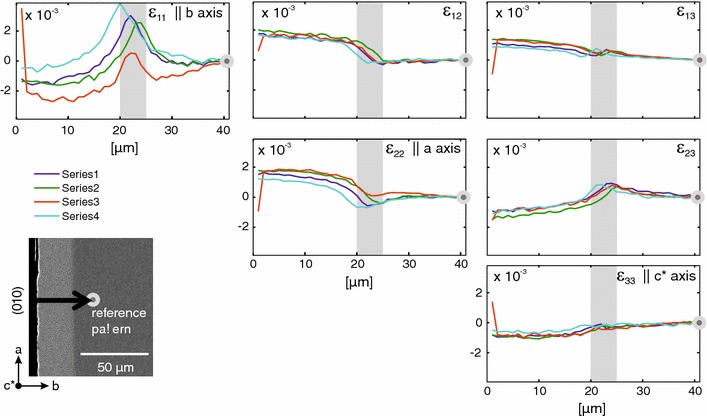
Above diagonal part of the symmetric strain tensor for profiles measured across a sharp diffusion front; the differently coloured profiles represent different measurements executed to test reproducibility, the reference pattern is located at the *right hand side* as represented by the *grey dot* and the edge of the crystal is on the *left hand side*; the most notable features are the strain concentration parallel to *b* within the diffusion front, and the extension parallel to *a* within the exchanged rim

Using the cross-correlation method, the full strain tensor was determined for all measured points along the profile. As the tensor is symmetric, only the above diagonal part is shown in Fig. 6. The profiles show the distortion of the lattice relative to the reference pattern. The diffusion front is located at a distance of about 20 μm from the polished (010) surface of the crystal and is about 5 μm wide. Four different profiles were measured to test for reproducibility. Except for some minor variation for $\epsilon _{11}$ the different measurements were generally in good accordance. The slight offset between the different series is probably a consequence of the preparation of the plates. The edge of the crystal is slightly rounded during polishing so that it is hard to find the exact position of the trace of the crystal surface, which defines the starting point of the profile.

The variations of the normal strains are most significant. The longitudinal strains along the *a*- and *b*-axis of the crystal, that is $\epsilon _{22}$ and $\epsilon _{11}$, respectively, show the most pronounced variations. The variation of $\epsilon _{22}$ along the profile indicates a continuous extension of the lattice in *a*-direction within the exchanged rim relative to the reference pattern. The extent of the extension decreases inwards from the sample surface and vanishes at the diffusion front. In contrast, $\epsilon _{11}$, i.e. the strain in *b*-direction, is close to zero in both the rim and unexchanged core, but indicates a localised extension in *b*-direction at the diffusion front. Within error, the values stay constant for $\epsilon _{33}$ all along the measured profile indicating that no significant longitudinal strain occurred in c*-direction.

$\epsilon _{12}$ shows the same trend as $\epsilon _{22}$ with a slight rise in the exchanged rim which then declines within the diffusion front. $\epsilon _{23}$ shows the opposite trend with values being slightly lower in the rim relative to the reference pattern before rising in the diffusion front. None of the profiles shows any specific trend within the diffusion front itself. Within error, the values stay constant for $\epsilon _{13}$ all across the profile.

**Fig. 7:**
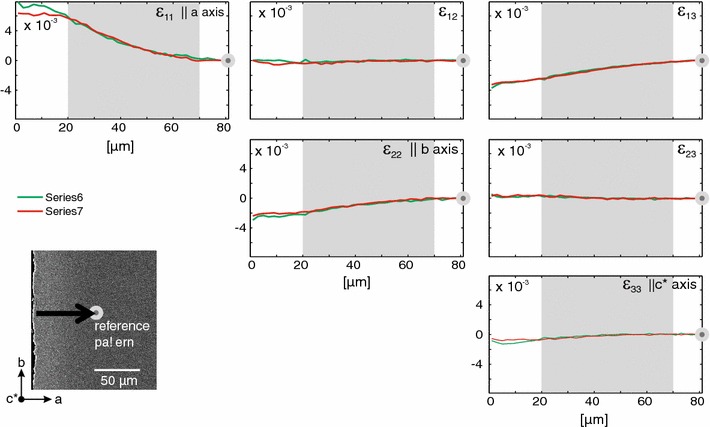
Above diagonal part of the symmetric strain tensor for profiles measured across a broad diffusion front; the differently coloured profiles represent different measurements executed to test reproducibility, the reference pattern is located at the *right hand side* as represented by the *grey dot* and the edge of the crystal is on the *left hand side*; in contrast to what was observed for narrow diffusion profiles, there is no strain concentration within the diffusion front, and a gradually increasing extension in *a*-direction is the most significant change observed

Two profiles were measured parallel to the *a*-direction of the same sample (Fig. 7). The diffusion fronts in this direction are much broader than those evolving parallel to *b*. The exchanged rim is about 20 μm wide, and the diffusion front has a width of about 50 μm. For these profiles, the longitudinal strain in *a*-direction $(\epsilon _{11})$ is characterised by a gradually increasing extension from the internal regions of the grain across the diffusion front and towards the exchanged rim. For the *b*-direction, we see the opposite trend with a gradual, slight contraction across the diffusion front. $\epsilon _{33}$ again stays roughly constant across the entire profile indicating that no significant longitudinal strain occurred in the c*-direction.

## Discussion

EBSD combined with the cross-correlation method is a powerful tool for quantifying coherency strain in the range of 10^−4^ (Maurice et al. 2012; Britton and Wilkinson 2012) provided the misorientation between test and reference pattern is limited to about 1°. A systematic error analysis of the cross-correlation method has shown no significant difference in accuracy between normal and shear components.

For the present measurements, the strain components in *y*-direction ($\epsilon _{22}$, and $\epsilon _{12},\, \epsilon _{32}$) will most probably show a reduced accuracy, because the patterns have been acquired by integration of an area extending 50 μm in *y*-direction. This corresponds to a horizontal blur of about 1 pixel in the patterns. However, as the patterns are changing only in *x*-direction of the sample, this horizontal blur stays constant for all measurements and should therefore not strongly reduce accuracy.

In the investigated feldspars we find lattice strain localised across a sharp diffusion front propagating in *b*-direction (Fig. 6). While the composition of the feldspar changes in a strictly monotonic manner with an outwards increase of $\hbox {X}_{\mathrm{Or}}$ from 0.85 to 1.00 across the diffusion front, the observed lattice strain across the sharp composition front is characterised by a substantial extension of the *b*-parameter that is localised to within an about 5 μm wide domain around the diffusion front. At the same time, the *a*-parameter shows a slight and monotonic contraction, and the *c*-parameter remains unchanged. This systematic change of lattice strain does not confer to the change of lattice strain which would occur in a stress-free crystal of alkali feldspar subject to a similar composition change. When alkali feldspar with an initial composition of $\hbox {X}_{\mathrm{Or}} = 0.85$ is shifted to the pure potassium end-member composition, $\hbox {X}_{\mathrm{Or}} = 1.00$, this implies an expansion in all crystallographic directions; $a_0 = 8.545$ Å$\rightarrow a_1 = 8.6$ Å, $b_0 = 13.025$ Å$\rightarrow b_1 = 13.03$ Å, $c_0 = 7.175$ Å$\rightarrow c_1 = 7.18$ Å, and a minute change in the angle $\beta$, $\beta _0 = 116.0^\circ \rightarrow \beta _1 = 116.05 ^\circ$ (Kroll et al. 1986). The temperature dependence of the lattice parameters is comparatively small and shows the same trend, with the *a*-parameter changing by about 1 % and the *b*- and *c*-parameters only by about 0.05 % in the temperature range between 20 and 1,000 °C (Henderson 1979) so that its effect can be deemed negligible in the analysis at hand.

**Fig. 8:**
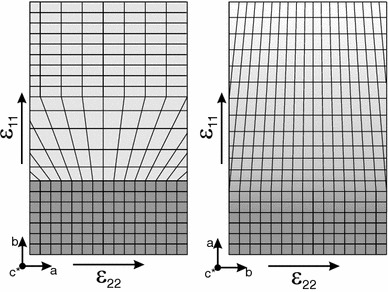
Schematic diagrams of the lattice distortion across the sharp (*left*) and broad (*right*) diffusion fronts propagating parallel to the *b*- and *a*-directions, respectively; the *dark grey colour* represents part of the unexchanged core, and the *light grey* represents part of the exchanged rim; for the sharp diffusion front, the lattice dilates in *b*-direction as (010) is the only free surface, while the *a*- and *c*-parameters are dominated by the unexchanged substratum, in the rim the dilatation in *a* which would be expected in a stress-free feldspar takes over and the gradual extension in *a*-direction observed for the broad diffusion front in turn is in accordance with composition strain in an unstressed feldspar

Within the plane of the diffusion front, that is, within the contact plane between the compositionally distinct domains of the crystal, the lattice parameters are confined to those of the volumetrically by far dominant internal portion of the crystal in which the original composition is retained. As a consequence, the volume change across the diffusion front occurs via change of the only unconstrained lattice direction (here the *b*-direction), perpendicular to the constrained interface. Only a few micrometers outside from the diffusion front, the mechanical coupling to the rigid substratum becomes less strong, and the crystal can expand in *a*-direction (Fig. 8, left). As soon as the composition strain is accommodated by extension in *a*-direction, the *b*-parameter shrinks again, leading to a strain similar to what is expected in a stress-free crystal of alkali feldspar.

The systematics of the strain along the profile taken across a broad diffusion front propagating parallel to *a* confers to what is expected for the composition induced eigenstrain in a stress-free feldspar. The *a*-parameter increases successively towards the outer portions of the crystal as here the free surface is perpendicular to the *a*-direction which is thus not confined by the stronger substratum (Fig. 8, right).

The mechanical effects due to the eigenstrain associated with diffusion induced composition change may have an influence on diffusion. The eigenstrain may interact with diffusion in two ways. On the one hand, it may change the thermodynamic driving force for diffusion, and on the other hand, the energy barriers for atomic jumps may be modified by lattice distortion. In a theoretical study, Larche and Cahn (1982) showed that the diffusion induced self-stress has an influence on the driving force for diffusion, which they expressed as the gradient in a generalised diffusion potential. This theory was extended by the consideration of the interplay between vacancy generation and annihilation and stress by Stephenson (1988). A more general concept for the kinetics of diffusion in a stressed solid was introduced by Svoboda et al. (2006). In this work, a distinction is made between interstitial and substitutional components and vacancies, and the role of non-ideal vacancy sources and sinks as well as the role of stress are addressed. The concept is based exclusively on the knowledge of the tracer diffusion coefficients and of the thermodynamic state functions of the bulk phase. As the shape of the diffusion fronts shows marked direction dependence, we do not think that the thermodynamic effect is the primary link between diffusion and self-stress. We rather think that the diffusion process is influenced via the effect of lattice distortion on atomic jumps.

The distortion of the lattice across the sharp diffusion front in *b*-direction may have an influence on the diffusion pathways in the feldspar structure. The main diffusion pathways are in the *a*–*c* plane. The alkali cations occupy large cavities within the Si- and Al-tetrahedral framework. The most likely interstitial sites for the large cations are $(0,0,\frac{1}{2})$ and equivalent sites, while no interstitial sites are available for diffusion in *b*-direction (Petrović 1972). For unit jumps parallel to the *a*-direction, the space between the atoms constituting the framework is large enough to allow the alkali cations to jump to the interstitial sites without the need for severe distortion of the lattice (Petrović 1972). Parallel to the *b*-axis, the cation sites are separated by the crankshaft-like chains formed by the Si- and Al-tetrahedra (Ribbe 1983), and the atomic jumps in this direction are more difficult. The extension in *b*-direction that is localised at the diffusion front corresponds to an increase in the length of the atomic jumps that the alkali cations contributing to diffusion in *b*-direction have to execute. It is conceivable that an increase in the jump distance between two neighbouring sites that are aligned in *b*-direction in the alkali sublattice goes along with an increase of the energy barrier between them. This would correspond to an increase in the activation energy for diffusion and would thus entail a reduction of the corresponding diffusion coefficient. Similar to the lattice distortion, this effect would be localised at the diffusion front and contribute to further sharpening of an emerging diffusion front. This feedback of diffusion induced lattice strain into the diffusion process itself may be one of the reasons for the formation of the exceptionally sharp diffusion fronts normal to (010) for shifts towards more potassium-rich compositions. In the case at hand, the self-sharpening diffusion front acts as a diffusion barrier, a feature that might be of interest in domain single crystals.

## Conclusions

We document the evolution of sharp concentration fronts during sodium–potassium interdiffusion in alkali feldspar in *b*-direction. In contrast, the concentration profiles are comparatively broad in directions contained in the *a*–*c* plane. The extraordinarily sharp composition gradients in *b*-direction only occur at $X_\mathrm{Or}>$ 0.95 and do not confer with theoretical predictions from interdiffusion models. The sharp composition gradient is related to a sharp lattice distortion gradient as measured using EBSD combined with a pattern cross-correlation method. The phenomenon of the extraordinarily sharp composition gradients is ascribed to the combined effects of the pronounced composition dependence of the interdiffusion coefficient and the distortion of the feldspar lattice in response to chemical eigenstrain. The lattice distortion due to chemically induced coherency stress may act as a self-induced diffusion barrier. This phenomenon has potential applications in designing domain single crystals with custom tailored transport properties.
